# Transitions to adulthood: self‐governance and disciplining in the making of patient citizens

**DOI:** 10.1111/1467-9566.13019

**Published:** 2019-10-30

**Authors:** Alicia Renedo, Sam Miles, Cicely Marston

**Affiliations:** ^1^ Public Health, Environments and Society Faculty of Public Health and Policy London School of Hygiene and Tropical Medicine London UK

**Keywords:** healthcare transition, identity, self‐governance, sickle cell, transition to adulthood, young people

## Abstract

Young people develop new behaviours and redefine their identities during health transitions when they move from paediatric to adult healthcare environments. Their identities help to guide their health‐related actions in response to life changes. Young people's health is increasingly recognised as important, yet we lack understanding of how health transitions shape identities and how they relate to other transitions to adulthood. We conducted a longitudinal interview study with young people with sickle cell disease to explore how young people define new identities as they transition to adulthood. We show how ‘disciplining at a distance’ via healthcare self‐management discourses and neoliberal norms governing adolescence play out in the tensions participants encounter when they are crafting new identities. Health transitions involve struggles to negotiate competing demands for self‐discipline. It is crucial to create enabling spaces for young people to protect their health while still developing identities that help them achieve life goals.

## Introduction

Young people's health and transitions to adulthood are now a global priority area for action (e.g. World Health Organization 2017). Lifelong health‐related habits develop during transitions to adulthood (Hagell *et al*. 2017) and various healthcare transition initiatives and guidelines are being introduced to support adolescents and young people to develop self‐management skills and health‐related knowledge (e.g. NICE 2016; World Health Organization 2017).

Health transitions require not only new behaviours but also new self‐perceptions and understandings of how one should behave as an adult patient (Viner 1999). Adolescents’ and young people's needs are a priority, yet we lack understanding of how health transitions shape their identities – that is, who they are and how they should be and behave as adults – and how this contributes to how they develop into adult patients. Self‐perceptions play a key role in shaping individuals’ orientation towards health (Jovchelovitch and Gervais 1999, Tarrant *et al*. 2012). A focus on how identities develop during transitions can help explain young people's success or otherwise in moving into healthy adult life. Here we explore how young people living with chronic illness craft new identities as they transition to adulthood. In doing so, we contribute to theorisations of the ‘making’ of adult patients during health transitions.

## Complex transitions and identity work

Transitions to adulthood are complex and non‐linear processes that involve developing one's identity (Arnett 2004, Renold and Ringrose 2011). The changes brought about by transitions create ‘ruptures’ in young people's existing habits, systems of knowledge and self‐definitions which require them to redefine their identities and understandings so that new ways of acting can emerge (Zittoun 2006: 6). Transitioning to adulthood involves re‐constructing for oneself both social categories such as ‘adult’, or ‘patient’, and related systems of values and ideas permeating our sociocultural and material environment (Duveen and Lloyd 2013). These available social categories, related values and ideas serve as frames of reference and semiotic rule systems for meaning making that young people renegotiate to develop self‐images that support their participation and regulate their behaviours in new contexts (Duveen and Lloyd 2013). In this way, identities are the products of and mirror ‘individuals’ efforts to situate themselves in their society’ (Duveen and Lloyd 2013: 159) as well as their efforts to respond to normative ideas (such as patienthood, ‘healthy lifestyles’), practices, others’ demands (from parents, doctors) and collective voices (such as educational systems, healthcare services) (Hermans 2001). How identities develop during health transitions can thus shed light on the making of adult patients and the adaptation to chronic illness by illustrating how young people re‐position themselves in relation to their health in order to work through life transitions.

The images we develop of ourselves and of our place in society through interactions with others, and the socially negotiated identities that emerge out of these encounters, play a key role in shaping health practices (Jovchelovitch and Gervais 1999, Marston *et al*. 2018). A nuanced exploration of transitions that takes into account the identities young people develop as they move into adulthood is vital to improve understanding of their success or otherwise in developing images of themselves that help them to engage in protective practices and function effectively across different contexts. Studying identity work is thus not only important to understand young people's experience of working through transitions (Wierenga 2011), but also to help improve healthcare transition initiatives aiming to support young people as they move into adulthood.

Discourses around transitions between paediatric and adult healthcare, in direct contrast with the sociological literature on transition to adulthood, seem to imply a linear (rather than ‘messy’) model of development: a smooth movement into independence (Allen and Gregory 2009) or movement into a healthy lifestyle and economic productivity (e.g. World Health Organization 2017). This model seems to be underpinned by a normative characterisation of adolescence as a vulnerable and problematic phase (Morrow 2013) that simply needs to be addressed through adequate transitional care (Allen and Gregory 2009). Conceptualisations of linear health transitions co‐exist with linked preoccupations about young people not adhering to treatment, or disengaging from services (e.g. Department of Health 2006). This type of representation of young people as potentially ‘at‐risk’ (Kelly 2001) invokes the idea of the need to shape ‘youthful identities’ (Kelly 2001) into particular types of adult patients, and transitions as an opportunity to fix reckless, non‐adherent and disengaged adolescents, so that they become independent, compliant and rational adults who take control of their condition (Allen and Gregory 2009). There is an implication that adult‐like rationality will translate into healthy behaviours (Allen and Gregory 2009). Yet multiple interconnecting factors – including, crucially, identities – shape young people's understandings of and responses to health issues. Identities are socially produced and negotiated in response to life changes including transitions; they function as ‘recipes for living’ or ‘interpretative frameworks’ through which health behaviours and new experiences are mediated (Campbell 1997: 274).

In this article, we adopt a relational view of health transitions, viewing transition to adulthood in general as a fluid, messy process in which identity work is central (Arnett 2004, Durham 2017, Renold and Ringrose 2011) and in which young people develop adult patient identities while also negotiating how these relate to their other evolving identities (e.g. student, adult). In this transition process young people must situate themselves in relation to available discourses (such as how adolescence is socially represented), social positions (such as the position as adult patient) and social categories of adulthood and health (such as healthy lifestyle, self‐management). We take a dialogical approach to understanding identity (Hermans 2001, Hermans and Kempen 1993, Marston *et al*. 2018, Renedo 2014), attending to its relational nature, rather than seeing identity as a static property of an individual. In this dialogical conceptualisation of the self (Hermans 2001, Hermans and Kempen 1993, Salgado and Hermans 2005) identity is developed through and against a person's repertoire of relationships with multiple others and the ‘polyphony’ (Bakhtin 1984) of their voices, assimilating and negotiating subject positions, discourses and social expectations made available to them through these social encounters. This perspective assumes that identity can thus be plural and contain contradiction (Renedo 2010, 2014) as it evolves in dialogue with others (present or imagined), through the different positionings of the self in response to others’ perspectives and demands (through agreement, disagreement, reformulation and/or tensions) (Hermans and Dimaggio 2007, Hermans and Kempen 1993). A dialogical approach to identity offers a way to examine how the discourses and multiple interactions between young people and others during transitions to adulthood constitute the relational context in which young people living with chronic conditions organise their transition experiences and develop a sense of self. This approach also helps us acknowledge the ‘elusive’ nature of adulthood (Durham 2017), as emerging and ‘in the making’ through continuing development of identities and ways of being (Renold and Ringrose 2011). Through this dialogical approach we examine the making of ‘youth at‐risk’ (Kelly 2001) into ‘governable subjects’ (Rose 1999b) who take individual responsibility for their own health and future life. We illustrate how the disciplining ‘at a distance’ (Miller and Rose 1990, Rose 1999a) of young people operates through the way young people shape their own identity. We show how the process of healthcare transitioning functions as a governing force on young people and involves a movement into self‐disciplining and modes of patienthood that are in tension with other emerging identity positions.

We examine health transitions amongst young people with sickle cell disease, a genetic blood disorder which disproportionately affects minority ethnic communities in Britain (Dyson 1998), mainly Black African and Afro‐Caribbean ethnic minority groups (Hickman *et al*. 1999). Sickle cell disease is a chronic debilitating condition that causes cumulative damage to multiple organ systems and is characterised by unpredictable and episodic acute painful episodes (also known as ‘pain crises’) that can be excruciating, require timely and adequate treatment (NICE 2012) to avoid complications (Wilson and Nelson 2015). Self‐management of sickle cell disease is demanding and is instilled as a form of ‘self‐regulatory governance’ (Dyson *et al*. 2011: 470). Self‐management practices include: keeping hydrated (drinking water), keeping warm, resting, avoiding arduous physical activity and being attentive and responsive to signs of a possible painful episode (Oni *et al*. 2006: cited in Dyson *et al*. 2011). Apart from a few exceptions of key sociological work (e.g. Atkin and Ahmad 2001, Dyson *et al*. 2010, 2011, 2016) there is a paucity of research into the lived experience of sickle cell (Dyson 1998).

## Methods

This was a co‐produced, longitudinal study (Miles *et al*. 2018). We interviewed young people aged 13–21 with sickle cell disease about their experiences of navigating health transitions beyond clinical settings to consider the wider context of young people's lives. The age range was designed to capture the gradual process of healthcare transition; from the time when the concept of transition is introduced in healthcare services to the post‐transfer period in adult services.

In total, we conducted 80 in‐depth interviews with 48 young people in England (30 young women and 18 young men, aged 13–21). Of these, 27 were one‐off interviews (17 with 19‐ to 21‐year‐olds, and 10 with 13‐ to 18‐year‐olds) and 53 were repeated interviews with twenty‐one 13‐ to 18‐year‐olds with whom we met 2–3 times over a period of approximately 18 months.

Participants were recruited via hospitals and outside healthcare services via our network of contacts with patient advocates. We designed youth‐friendly research leaflets and tried to conduct most interviews on participants’ ‘home turf’. AR conducted interviews at a location of participants’ choice, usually their homes and sometimes healthcare settings. We explained that we were independent researchers who were outsiders to their healthcare services context.

Topic guides were informed by discussion with service user representatives in the project (Miles *et al*. 2018). Interviews explored lived experiences receiving health care and social aspects of interviewees’ lives. Repeated interviews gave us the opportunity to explore experiences over time and capture the evolving and unpredictable nature of sickle cell disease (Fuggle *et al*. 1996). The development of each individual's topic guide for follow‐up interview was informed by previous interviews. Interviews lasted between 60 and 90 min, were audio‐recorded and transcribed verbatim. Participants were given information about external agencies they could contact if they needed help with issues raised in the interview.

We analysed interviews using an inductive, iterative approach, following some of the practical steps of Grounded Theory (Charmaz 2006), developing the coding frame inductively from the entire data set (Charmaz 2006), and refining it alongside data collection. We refined analytical categories during repeated rounds of coding and ‘memo‐writing’ (Charmaz 2006) (about codes and emerging analytical themes), and via reflective analytical sessions with healthcare service user representatives in the project. In the analysis we attended to the types of subjects being produced through the narratives. We focused on how ‘things said’ made certain subject positions available and produced certain kinds of subjects at different points of the interview (Bacchi and Goodwin 2016: 116). Our dialogical approach (Aveling *et al*. 2015, Renedo *et al*. 2018) helped us pay attention to the contextuality of language (Linell 2009, Marková 2003) to move beyond speech/text as abstract reified forms (e.g. morphemes, syntactical structure) independent from social relationships and the outside world. Informed by Bakhtin's ideas about dialogue (Bakhtin 1981, 1984, 1986) and the dialogical production of identity (Hermans 2001, Hermans and Kempen 1993, Renedo 2010), we focused on how participants accounted for their experiences in a relational way, through and against others (parents, healthcare professionals, peers) including the researcher, and positioning themselves in response to their voices. We focused on utterances in the context of the whole interview – by examining how utterances were interconnected with each other (i.e. how one utterance qualified a preceding one, by for instance, investing it with new meaning, reformulating it or denying it). We also examined utterances in relation to the wider context of the research and young people's lives to understand the possible social origin of the dialogues refracted through interview talk (Aveling *et al*. 2015) – how meanings were articulated through different voices – and examine the social ‘genealogy’ (Bacchi and Goodwin 2016: 116) of the subject positions taken by participants. After rounds of grounded analysis we identified the theme of taking responsibility for oneself. At this analytical stage we used literature on governmentality (Miller and Rose 1990, Rose 1999a,b) to help refine the emerging theory on identity. We also examined the data reflexively with attention to our role in co‐producing the data as white, academic researchers without this health condition and how we might have helped shape the subject positions taken by participants. The study received ethics approval from the London School of Hygiene & Tropical Medicine and NHS ethics committees (REC 15/LO/1135).

## Findings

With our first theme, we illustrate how authoritative healthcare self‐management discourses discipline young people ‘at a distance’ (Miller and Rose 1990, Rose 1999a) permeating the way young people work on themselves to shape their behaviour to align with these self‐management discourses. Under our second theme, we examine how disciplining at a distance plays out in identity work in which co‐existing self‐disciplining positions compete with one another, as disciplinary social norms governing adolescence become entangled with health self‐management discourses.

## Healthcare transitions: the push for self‐discipline and self‐improvement

Interview accounts illustrate how disciplining at a distance works through how young people ask themselves to govern their bodies and take responsibility for their illness. Participants present themselves as needing to improve and become more responsible. The work of disciplining at a distance emerged in the form of participants addressing themselves in talk, problematising their own behaviour, asking themselves to take individual responsibility and to transform into particular types of subjects:

The reasons like I'm ill […] is ‘cause I wasn't really paying attention to the way I was feeling. […] I could have prevented it [pain crisis] if I'd paid attention […] I should pay attention to this. ‘Cause, yeah, attentiveness, that's the thing **I need to work on** […] I think I'm slowly getting there […] like trying to change it […] I am paying more attention in everything, really. (E4, 19–21 years old)

E4's quote illustrates the type of proposals for individual change contained in many participants’ accounts. Participant E4 asks herself to learn how to listen to her own body and take responsibility to avoid pain crises. When envisaging a healthy future later on in the interview, she draws on individualising discourses – talking about working on herself to improve habits and self‐management practices, for instance. Similarly participant I5 reminds herself to monitor body temperature and energy levels to avoid a pain crisis:

**I have** to try and be, yeah, **you have** to try and like relax more and […] Like not use a lot of my energy and like keep drinking water and stay away from like heaters […] (I5, 13–15 years old)

In calling on themselves to self‐discipline, participants were likely echoing the self‐management discourses they hear from carers and healthcare professionals. Participants’ accounts were individualising, locating control of health and of moving into healthy adult life within a purposeful and agentic self who makes the right choices and governs own life. When we asked how they could be supported, their responses did not generally refer to others. Instead, they framed transitions as an issue to be managed by the individual. Successful transition to adulthood requires acting on oneself: ‘starting to help myself […] knowing how to take care of myself’ (I5). Healthcare transition was narrated as an individual change through which one develops knowledge of one's own body and actively monitors it, regulating behaviour to maintain health. Healthcare transitioning as a movement into self‐disciplining and self‐governance is learned through therapeutic relationships with doctors/nurses and echoed within family relationships. Adults’ reminders about the punishing consequences of not taking responsibility, and the internalised adult gaze act to discipline young people at a distance.

I didn't care that much, um, but then […] as you get older you know that you really need to start doing it [taking medication, attending appointments] […] sometimes you get tired and you like you don't want to take any medication, and you don't want to go into hospital, um but then when they [doctors/nurses] tell you **what the consequences** will be, you get to understand more […] she [nurse] told me what will happen if I don't take it and how […] there's a possibility I could die, and then she said I won't be able to [practise favourite sport] and so when I heard that […] I thought, OK, I have to start taking it then […] I've just mostly learned about how to keep my ferritin levels down [….] they check it at hospital so they'll know if I've had medication or not. And so it makes you think, oh they're going to find out [that I am not taking medication] anyway, so […] oh I should just grow up and do it […] And just take my medication and just go to the hospital, because **it's only helping you.** (O7, 13–15 years old)

Participant O7 (above) said he found it difficult to engage in desired self‐management behaviours, but draws on someone else's voice (presumably the nurse) addressing him in talk to remind himself about the type of subject he could become, and delineating how he ought to be. Participants talked about being constantly reminded by parents to stay hydrated and to wear warm clothes to avoid pain episodes. Although adult reminders could be annoying, the adult gaze worked as a reminder ‘to help me to stay healthy’ (E4, 19–21 years old) encouraging participants to be of a particular type of patient. After a long stay in hospital, participant I8 (13–15 years old, below) decided to start to ‘just take care of myself now’ (I8). Parents’ reactions and comments about an unhealthy lifestyle might have helped her internalise a sense of individual responsibility for the consequences of her choices:

My [mum/dad] was just telling me, you probably didn't drink water, you probably didn't do your exercise. ‘Cause I probably wasn't drinking water ‘cause, um, the only thing I would drink is like fizzy drinks […] I tend to drink a lot of water now […] that's the key. (I8)

## Managing interdependent and juxtaposing identities

Individualising healthcare self‐management discourses that govern young people at a distance play out in the way participants defined their patient identities and developed self‐disciplining subject positions. Healthcare self‐management discourses become entangled with wider disciplinary discourses governing adolescence and transitions to adulthood. This plurality of discourses plays out in participants’ identity work and translates into tensions between self‐positions that are difficult to reconcile: their disciplined patient self and their self‐actualising self who wants to meet educational/career goals.

### Disciplined patient self

Participants talked about being, and working on being, disciplined in self‐management. They talked about monitoring their bodies (temperature, types and levels of pain), drinking water, avoiding cold weather and wrapping themselves up to keep warm. They talked about having developed techniques to self‐manage a pain crisis, being responsible with pain relief and rationing it to ensure its effectiveness. They also talked about organising their everyday life carefully around their self‐management and education or work needs.

I always have water and my medicine in my room […] I don't go out as often, I've realised that, um, I like to study more […] I know how to keep myself warm with clothing and stuff before anything else […] I arrange my sleeping patterns in a way, like I'm getting enough sleep and stuff. So right now I'm trying to prevent feeling really tired and having fatigue […] since I was younger they've always told me like water is the main thing […] So I'm keeping hydrated and stuff […] I just try and do it [read up on sickle cell disease] as much as possible so I'm learning about it each time […] so I understand. (A6, 16–18 years old)

For young people transitioning to adulthood and adult healthcare services, self‐disciplining involves being committed to learning more about themselves, acting on their own bodies and making the ‘right’ healthy choices. It also involves self‐regulating their everyday lives temporally and spatially to avoid or manage crises (Z1 & U10 below). Young people had, and reported increasingly developing, a temporal sense of their condition and bodies, of the timing of pain events and of the temporal outcomes (e.g. length of recovery) of different types of pains. They closely monitored these temporal aspects and regulated self‐management practices to control outcomes (e.g. acuteness of crisis) so that they could resume school or work responsibilities as soon as possible. Participants preferred to manage a crisis at home because, they said they could rest and recover more quickly there. Over time they had learned about the importance of acting quickly to avoid a crisis escalating in order to avoid hospital admission, which would affect educational performance or disappoint work colleagues. They regulated their behaviour spatially, for instance by keeping safe distances from home and hospitals when choosing where to go out, or where to attend university.

I have to be aware, just in case I do get sick. Cause sometimes in the day it could also just hit me […] So I have to be just like aware, to make sure that I don't overdo myself, or I don't walk too much […] especially in like really hot weathers or really cold weathers, so I have to make sure to like not go out for too, for a really long period of time. And also to always drink water […] to keep myself hydrated […] I try to make sure that I'm not going out too, too often, or if I do go out with my friends I'd make sure to go around [the neighbourhood] and not too far away, and make sure that my mum knows exactly where I am in case I do fall ill, then she'd be able to pick me up. (Z1‐ 16–18 years old)

Participant Z1 positions herself as an expert in her own body, actively listening to its needs to ensure she does not push body limits. She knows the different types of pain and the types of acute pain episodes they would lead to, and assesses whether she needs to go to hospital. Socialising is another aspect of Z1's life that needs to be carefully regulated. Participants often told us how they planned carefully ahead for social outings, thinking about travel arrangements and mapping activities well in advance to ensure quick access to healthcare services if needed. They also said they avoided certain activities or found ways to navigate them to avoid feeling unwell, for instance by knowing where there was a place to rest, or whether there was a lift they could use to avoid fatigue. The disciplined patient limits social activities to stay healthy and this shapes the type of social self the young person can become. Participants aligned themselves with ways of being that enabled them to self‐manage, constructing social identities that distanced them from their healthy peers who liked socialising. They would present themselves as ‘quiet’, ‘not outgoing’, as liking staying at home and in some cases as more mature than their peers. Participant U9 (19–21 years old) told us he did not like football and clubbing anymore. He preferred to stay in because he did not ‘want to damage’ his body. Similarly Z3 (19–21 years old), explained he would not go out in the winter, distancing himself from his University peers who ‘go out partying’ explaining he is ‘not one of those people anyway’.

Young people's narratives contained discussions about the challenges of trying to reproduce autonomous and self‐sufficient adulthood, including – for older participants – wanting to move out of their family home. For some, leaving home and the journey to become a disciplined patient was difficult, particularly without parental support:

I'm not as nice to myself as I was treated back when I was living with my parents. […] You have to learn how to do it yourself […] think that's why I leave it till it's really bad [the pain] […] if I was at home my mum […] would be like, hey, yeah just leave it. Like, just calm down, take some painkillers until it subsides […]. But here I'm like, yeah I'll be fine, I'll keep on working. (I9, 19–21 years old)

Three participants presented themselves as not having made the move into full self‐management. They explicitly distanced themselves from their illness and told us about not taking their daily penicillin and folic acid. They said they did not need supplements because they were ‘fine’, and/or because they could not see any benefit from taking them. Yet later on in the interviews two participants specifically talked about not taking supplements as a reflection of ‘ignoring’ their condition (I6, 19–21 years old) and of trying to preserve a sense of being normal (E1 below).

[I don't take the supplements] because I think sometimes I think I'm fine, so there'll be a period may, for a year, I don't have any crises […] And I'll just think I'm normal. I'm fine, (.) that my body can cope but really I don't think it can […] it's not that taking medicine is difficult it just sometimes reminds you that you're sick so it makes me sad.[…] I really should take medication […] I should really tell my doctor (E1, 19–21 years old)

### Being self‐actualising and productive versus self‐managing my condition: The ‘Lazy’ Self

Disciplining oneself extends beyond health management into education and work. Participants echoed the discourses of self‐efficacy and personal achievement typically articulated within educational environments. Participants presented themselves via narratives of self‐improvement (trying to do my best) and productivity (being active). Participant A6 (above) not only took individual responsibility for self‐management, but also presented herself as responsible in her studies and in learning about sickle cell. Likewise, participant Z3 positioned himself as self‐sufficient in self‐care and knowledgeable about his condition. Earlier in the interview he said he had achieved the grades he needed for his degree despite having had time out because of his sickle cell. Yet he said that it ‘wasn't the best [he] could have done’. He ‘wanted to do better for [himself]’. Adopting this tough position towards himself might be indicative of adults’ disciplining at a distance and attempts to shape young people into certain type of adults. He later mentions ‘always get[ting] drilled in like: we need to have good GCSEs, need to have good grades’. Participant U10 (below) said he listens to his own body to minimise the impact of sickle cell on his education.

[…] if I'm sick I won't have any school at all. So it's better off missing one day than missing all the lessons […] I know what I have to do to keep myself well […] everything they tell [doctors, nurses] me to do, like get sleep or do this, I just do them, because I know it's crucial for me. (U10, 13–15 years old)

Being a responsible patient who rests or stays at home to self‐manage is in tension with the self‐actualising self who wants to work hard to develop and perform well. The tension between these entangled identities is played out in young people's frequent characterisations of themselves as ‘lazy’. Disciplining oneself includes time management and regulating social interactions (e.g. avoid friends’ distractions during class) to stay focused and deliver work on time. Participant Z4 (below) positioned himself as self‐governing to achieve career goals and as more responsible than his university peers. This type of narrative performance, or real‐life strategy, might help him increase his distance from a ‘lazy’ self. Participant Z4 also talked about staying in hospital when he was younger. He characterises his preference for staying in bed and watching TV when being unwell as a ‘lazy’ choice over attending the hospital school. Young people's accounts emphasised resting as both ‘lazy’ and unproductive, while also recognising it as a key self‐management practice.

I have a, er, a big inspiration, which is to be successful in the future […] just keep on working hard [to achieve this] […] keep studying […] I like t**o focus and work a lot**, but sometimes [students during lectures] will, like, lose concentration and do silly stuff […] (Z4, 19–21 years old)

I'm always feeling tired. So like when I'm feeling tired I, I don't like, I don't like doing nothing, so sort of I'm like lying in bed or go sleep. But, being, being in bed all day like it's not, it's like, it's just not, not, not good really, it's just like, being tired all the time, lying in bed like, you're not doing nothing, are ya? It's like, I wanna like try my best and not, like, not get into bed. (O2, 16–18 years old)

Note the dialogical elements in participant O2's quote above: ‘You're not doing nothing, are ya?’. This could be the young person addressing herself, or could also be interpreted as the young person imagining herself being addressed by someone else who might be seeing her as lazy. Young people worried about others seeing them as lazy and talked about being called ‘lazy’ (e.g. by friends or siblings). Participant O2 characterises resting as not an appropriate practice and she tries to act on herself to rectify it to become active. Interviews were permeated with accounts of having to rest or stay at home to manage fatigue. Yet at points participants shifted to positions which presented a more active self or a transformative self, working towards being as active as possible, to try to avoid being seen as lazy by others (participant U9 below).

Participants talked about getting ahead of a potential acute pain episode. They disciplined themselves to make the most out of their wellness periods by doing extra work, even if this involved pushing body limits (e.g. not taking time off work, working late at night) and missing out on having fun with friends (e.g. avoiding distractions during class). Their accounts about doing extra work presented a disciplined self, aware of time and self‐regulating their behaviour to compensate for missing study time or work when they were ill.

[…] usually, like I, I work really hard like, and I work really fast because a lot of the time in school I would be out for so long that I have to catch up. […] If I like missed a few days I don't want it to look like, like I'm not as useful to the team [at work].[…] I didn't want to feel like I was like taking, taking the piss or anything but it, I think again, it's like the laziness thing, it's like in my head maybe […] sometimes I'll be reluctant to take time off [from work] or I'll try, or I'll just take painkillers and go rather than, erm, not going. (U9, 19–21 years old)

When I'm in hospital and I wanna work, and I try or I get too tired and I can't, it's just a bit frustrating […] I take my books with me but if I'm getting too tired then I have to stop and not doing as much as I want to, and that's why it upsets me. (I7, 19–21 years old)

Participant I7's account conveys the relentless self‐regulation for achievement that was in young people's narratives, which here takes the form of efforts to produce work when she is unwell. This illustrates some of the difficulties participants encounter when trying to reconcile their responsible student/worker self with the patient self they say they ought to be. Disciplining oneself becomes crucial in efforts to reconcile these opposing positions. The active and self‐actualising self might have to push body limits and ignore bodily signs in pursuit of academic achievement and work productivity, but also to avoid the making of a lazy self. Another participant (U2, 16–18) talked about forcing herself to manage an acute pain episode at home rather than going to hospital because she had a large amount of college revision to do. Others talked about how important it was for them to be productive members of society and distanced themselves from those who they saw as letting illness ‘hold [them] back’ by ‘sit[ting] at home’. Similarly, participant O1 talked about herself as being ‘lazy’ when feeling unwell, tired and wanting to stay in bed. Yet she ‘force[s]’ herself to be productive, helping parents with chores and going to school. Self‐regulating in this way is important for her to avoid developing what she sees as inappropriate habits and taking control over shaping the type of subject she wants to become:

There's been days where I don't do anything and then I'll get used to not doing anything, so I'll get into the habit of being lazy. (O1, 13–15 years old).

Cultivating the aspirational adult self requires working hard and ‘pushing’ oneself to get good grades to achieve life goals. ‘Pushing yourself’ is also indicative of the entanglements and tensions between the self‐actualising self and the patient self who is aware of the consequences that overdoing can bring to my health. The two self‐positions mutually shape each other.

I'm trying to work on my cardio vascular so I can, my heart can pump like oxygen round more. That will benefit me […] I'm more active this time, yeah. […] I've been researching a lot. […] I've been around going online, and stuff like, what type of fruit I could eat, that's really beneficial like, […] and like iron as well, iron deficiency, um, trying er, you know, make a, improve my health like health style, life style, so I'm trying to become more fitter […] the only way I could make improvement or continue going over crisis is by improving my body that's why I'm taking care of it […] I do try to push myself um, to do work [at college when feeling tired] but if I can't, I probably just relax, talk to some friends, stuff like that. Um, yeah, and then when break time comes I eat something, relax a bit and then sometimes I do have the energy to do some work, try and push myself and then later on I rest and then complete the work or try and finish all of it (I3, 16–18 years old)

In the first interview participant I3 talked about trying to control his tiredness and low stamina (‘I push myself’) when doing sports and how at times this had led to an acute pain episode (‘crisis’). At third interview, he presented himself as more in control of his health, devoted to cultivating fitness and self‐governing his healthy choices. He was transitioning towards a healthy lifestyle and was also disciplining himself to succeed in life. Participants talked about their frustration with getting tired and being unable to exercise when they wanted to become fitter. The boys and young men in particular talked about their need to work on themselves to improve body performance and stamina.

## Discussion

We have shown how crafting new identities during healthcare transitions is a dynamic process that involves young people conceptualising themselves as in need of improvement and struggling to negotiate competing demands for self‐governance from other intersecting life transitions they are undergoing simultaneously. Authoritative healthcare self‐management discourses and neoliberal norms governing adolescence more broadly discipline at a distance, enter into the making of young people's subjectivities and translate into interdependent and conflicting self‐disciplining identities. Young people in our study struggled to develop a unified, coherent sense of self as they were caught between competing yet interdependent positions as both disciplined patients and as self‐actualising and competent students/professionals. These difficulties can play out in a stigmatising self‐identification as ‘lazy’ and in individuals becoming involved in exhausting attempts to reproduce normative ways of being an adult. Teachers’ and peers’ stigmatising misrepresentations of sickle cell‐related fatigue as a quality of someone being lazy (Dyson *et al*., 2007; Dyson *et al*. 2010) may also contribute to this stigmatising self‐identification.

The complexities of formulating a coherent and consistent identity that young people experience (Renold and Ringrose 2011, Zittoun 2006), are rendered more complex for young people with sickle cell disease with the addition of healthcare transitions. For these young people, transitions to adulthood involve relentless self‐disciplining and self‐surveillance to produce bodily efficiency and maintain health, whilst also working hard to achieve and cultivate a self‐actualising aspirational self. Their self‐regulation goes beyond their body limits to expand into temporal, spatial and social aspects of their everyday lives. Each type of self they are trying to enact imposes demands that act as ‘pushes and pulls’ (Renold and Ringrose 2011: 392) in multiple directions. ‘Pushing yourself’ becomes an important tactic to meet education and work responsibilities as well as helping handle social expectations surrounding academic achievement.

Using a dialogical approach, we have illustrated how the development of new positions during transitions to adulthood is done through and against normative disciplinary ‘proposals’ for selfhood circulating in young people's social environments and permeating their relationships with adults. In this dialogical process of identity formation, there seems to be little room for young people with sickle cell disease to contest the discourses and demands posed by adult others. If they resist or fail to assimilate these competing expectations on the self, the risk is developing a stigmatising sense of personal laziness and irresponsibility. We have shown the identity processes through which individualising self‐management and ‘healthy lifestyle’ neoliberal discourses that characterise health care today, intersect with neoliberal demands from educational contexts (Fendler 2001, Jeffrey 2010) which ask young people to excel and become entrepreneurial, competent subjects. These intersecting neoliberal demands act as a form of disciplinary governance (Lupton 2013) by crafting identities ‘at a distance’ (Miller and Rose 1990, Rose 1999a) and identities that resemble the prototype of the neoliberal citizen (responsible, productive, competent, autonomous). For our participants, self‐care and self‐governance to become a neoliberal subject involves extensive and continuous effort to sustain an identity while maintaining their own health. The juxtaposition of their identities and the difficulties young people experience when trying to reconcile them, reflect the competing nature of these demands on the self. The crafting of identities ‘at a distance’ (Miller and Rose 1990, Rose 1999a) was evident in the dialogical nature of interview narratives; the way young people drew on disciplining discourses and on others’ voices to demand that they improve themselves and to present themselves as working to meet competing neoliberal demands. This making of young people into ‘governable’ (Rose 1999b) patient citizens was also evident in the way participants individualised their illness experiences and located responsibility for moving into a healthy adult life on a purposeful and agentic self.

A focus on identity formation and its relational nature can help explain ambivalence and tensions underlying transitions and the sources of young people's struggles as they try to develop self‐understandings and interpretative repertories to guide their health‐related actions as they move to adult services. This is particularly relevant for the case of young people with sickle cell disease who are ‘dys‐positioned’ between competing demands emerging from their relations with adults in healthcare services and school contexts (Dyson *et al*. 2011: 465). On the one hand, in healthcare services they are inculcated to protect health through self‐care practices such as self‐monitoring of body temperature and hydration, and on the other, school emphasises regular attendance, achievement, engagement in physical activities and non‐disruption of classes for water or toilet breaks (Dyson *et al*. 2011). Previous studies of young people with diabetes have found how similar tensions between moral demands on young people to exert control on themselves translate into multiple and conflicting self‐disciplining practices (Balfe 2007). Developing medical autonomy at the same time as developing autonomy as an adult more generally adds complexities to the experience of growing up with a chronic condition (Heaton *et al*. 2016).

The relational nature of identity work has consequences for the status we confer on the study data. We need to be mindful of the fact that the identity work presented through narratives is also contingent on the interview situation and is relationally co‐produced through the research relationship itself (Miles *et al*. 2018). As researchers, we made efforts to avoid being perceived as the same adults that relay disciplinary discourses in healthcare and educational settings. However, the interview situation itself might have invited young people to enact socially desired ‘modes of subjectification’ (Rabinow and Rose 2016). This might be why they presented themselves as responsible and working on developing/having evolved into disciplined forms of patienthood. Yet they seemed comfortable about asserting their patient expertise during the interview and spoke out about the types of care they wanted. This is an exception to the type of self they enact in healthcare and educational contexts, where they struggle to be heard by adults (Atkin and Ahmad 2001).

Our research is likely to apply to other chronic conditions. We have shown how the dialogical crafting of identities at a distance during transitions gives us a novel way of understanding young people's experiences of healthcare transitions. The tensions between competing subject positions, the self‐regulation and self‐disciplining for self‐improvement which our participants deploy and the way the spatially specific nature of self‐management impinges upon their transitions shaping the type of subject they can become, may well be felt by other young people living with chronic conditions.

While young people's health‐related knowledge and confidence to move into independent adult life is often the focus of mainstream healthcare transition work (e.g. NICE 2016), we would argue that young people also need to be supported to negotiate the complexities of their sometimes competing identities (patient and non‐patient), which may well determine how well prepared they are to move into adulthood. To do this, healthcare transition support programmes would need to help young people manage the conflicting responsibilities they may experience with respect to their health and other aspects of their lives (Allen and Gregory 2009). They also need to recognise that conflicting identity work emerging from these competing demands is relationally constituted through the multiple practices around the young person; this includes the healthcare transition support itself, as well as neoliberal public health and educational discourses and relationships with carers who may act as relays for all these discourses. All of these practices and discourses permeating young people's relationships help define what young people can be, and participate in the making of ‘the kind of person it is possible’ for young people to ‘become’ (Bacchi and Goodwin 2016: 120), and set limits on what they can become. Healthcare transition support programmes must also work with young people in participatory ways (Miles *et al*. 2019) to stimulate critical reflective dialogue (Freire 1990) and help them to understand and critique the social conditions through which their identity is constituted. Participatory work should also help young people renegotiate highly individualising views on health and transitions that narrowly focus efforts on self‐improvements and changes at the personal level. This participatory work is crucial if we are to support young people's agency to cultivate self‐specified and fulfilling identities rather than simply pushing them to conform to hegemonic forms of adulthood. Any work to support young people's healthcare transitions should also involve working with their wider social contexts, including schools (Dyson *et al*. 2014), to create enabling spaces for young people to protect their health while still developing adult identities that maximise their potential to achieve their life goals.

## Data Availability

The datasets generated and analysed for this study contain sensitive personal data that were collected from children and adolescents. These data will not be made freely available by depositing them in a publically available repository. However, we will accept legitimate requests to access the data; requests should be made to Professor Cicely Marston. All requests will be considered on a case by case basis.

## References

[shil13019-bib-0001] Allen, D. and Gregory, J. (2009) The transition from children's to adult diabetes services: understanding the ‘problem’, Diabetic Medicine, 26, 2, 162–6. 19236619 10.1111/j.1464-5491.2008.02647.x

[shil13019-bib-0002] Arnett, J. (2004) Emerging Adulthood: The Winding Road From the Late Teens Through the Twenties. New York: Oxford University Press.

[shil13019-bib-0003] Atkin, K. and Ahmad, W.I. (2001) Living a ‘normal’ life: young people coping with thalassaemia major or sickle cell disorder, Social Science and Medicine, 53, 5, 615–26. 11478541 10.1016/s0277-9536(00)00364-6

[shil13019-bib-0004] Aveling, E.‐L. , Gillespie, A. and Cornish, F. (2015) A qualitative method for analysing multivoicedness, Qualitative Research, 15, 6, 670–87. 26664292 10.1177/1468794114557991 PMC4641508

[shil13019-bib-0005] Bacchi, C. and Goodwin, S. (2016) Poststructural Policy Analysis. A Guide to Practice. London, England: Palgrave MacMillan.

[shil13019-bib-0006] Bakhtin, M.M. (1981) The Dialogic Imagination: Four Essays. Austin: University of Texas Press.

[shil13019-bib-0007] Bakhtin, M.M . (1984) Problems of Dostoevsky's Poetics (C. Emerson, Trans.). Manchester: Manchester University Press.

[shil13019-bib-0008] Bakhtin, M.M. (1986) Speech Genres, and Other Late Essays. Austin: University of Texas Press.

[shil13019-bib-0009] Balfe, M. (2007) Diets and discipline: the narratives of practice of university students with type 1 diabetes, Sociology of Health & Illness, 29, 1, 136–53. 17286710 10.1111/j.1467-9566.2007.00476.x

[shil13019-bib-0010] Campbell, C. (1997) Migrancy, masculine identities and AIDs: The psychosocial context of HIV transmission on the South African gold mines, Social Science & Medicine, 45, 2, 273–81. 9225414 10.1016/s0277-9536(96)00343-7

[shil13019-bib-0011] Charmaz, K. (2006) Constructing Grounded Theory. London: Sage Publications.

[shil13019-bib-0012] Department of Health and Department for Education and Skills (2006) Transition: getting it right for young people: Department of Health and Department for education and skills.

[shil13019-bib-0013] Durham, D . (2017) Elusive adulthoods: introduction In Durham D. and Solway J (eds.) Elusive Adulthoods. Indiana: Indiana University Press.

[shil13019-bib-0014] Duveen, G. and Lloyd, B. (2013) The significance of social identities In Duveen G., Moscovici S., Jovchelovitch S. and Wagoner B. (eds.) Development as a Social Process: Contributions of Gerard Duveen. Routledge Ltd: Oxon, pp 156–72.

[shil13019-bib-0015] Dyson, S.M. (1998) “Race”, ethnicity and haemoglobin disorders, Social Science & Medicine, 47, 1, 121–31. 9683386 10.1016/s0277-9536(98)00023-9

[shil13019-bib-0016] Dyson, S.M. , Abuateya, H. , Atkin, K. , Culley, L. , *et al* (2010) Reported school experiences of young people living with sickle cell disorder in England, British Educational Research Journal, 36, 1, 125–42.

[shil13019-bib-0117] Dyson, S.M. , Atkin, K. , Culley, L.A. , and Dyson, S.E. , (2007) The educational experiences of young people with sickle cell disorder: a commentary on the existing literature, Disability & Society, 22, 6, 581–594.

[shil13019-bib-0017] Dyson, S.M. , Atkin, K. , Culley, L.A. , Dyson, S.E. , *et al* (2011) Sickle cell, habitual dys‐positions and fragile dispositions: young people with sickle cell at school, Sociology of Health & Illness, 33, 3, 465–83. 21375541 10.1111/j.1467-9566.2010.01301.x PMC3084516

[shil13019-bib-0018] Dyson, S. , Atkin, K. , Culley, L. and Dyson, S. (2014) Critical realism, agency and sickle cell: case studies of young people with sickle cell disorder at school, Ethnic and Racial Studies, 37, 13, 2379–98.

[shil13019-bib-0019] Dyson, S.M. , Ahmad, W.I.U. and Atkin, K. (2016) Narrative as re‐fusion: making sense and value from sickle cell and thalassaemia trait, Health, 20, 6, 616–34. 27491942 10.1177/1363459316660861

[shil13019-bib-0020] Fendler, L. (2001) Educating Flexible Souls: the Construction of Subjectivity through Developmentality and Interaction In Hultqvist K. and Dahlberg G. (eds) Governing the Child in the New Millennium. New York: Routledge, pp 129–52.

[shil13019-bib-0021] Freire, P . (1990) Pedagogy of the Oppressed (New ed.), Harmondworth: Penguin.

[shil13019-bib-0022] Fuggle, P. , Shand, P.A. , Gill, L.J. and Davies, S.C. (1996) Pain, quality of life, and coping in sickle cell disease, Archives of Disease in Childhood, 75, 3, 199–203. 8976657 10.1136/adc.75.3.199 PMC1511691

[shil13019-bib-0023] Hagell, A. , Shah, R. and Coleman, J. (2017) Key Data on Young People 2017. London: Association for Young People's Health.

[shil13019-bib-0024] Heaton, J. , Räisänen, U. and Salinas, M. (2016) ‘Rule your condition, don't let it rule you’: young adults’ sense of mastery in their accounts of growing up with a chronic illness, Sociology of Health & Illness, 38, 1, 3–20. 26140336 10.1111/1467-9566.12298 PMC4758387

[shil13019-bib-0025] Hermans, H.J.M. (2001) The dialogical self: toward a theory of personal and cultural positioning, Culture & Psychology, 7, 3, 243–81.

[shil13019-bib-0026] Hermans, H.J. and Dimaggio, G. (2007) Self, identity, and globalization in times of uncertainty: a dialogical analysis, Review of General Psychology, 11, 1, 31.

[shil13019-bib-0027] Hermans, H.J.M. and Kempen, H.J.G. (1993) The Dialogical Self : Meaning as Movement, San Diego, Calif. London: Academic Press.

[shil13019-bib-0028] Hickman, M. , Modell, B. , Greengross, P. , Chapman, C. , *et al* (1999) Mapping the prevalence of sickle cell and beta thalassaemia in England: estimating and validating ethnic‐specific rates, British Journal of Haematology, 104, 4, 860–7. 10192451 10.1046/j.1365-2141.1999.01275.x

[shil13019-bib-0029] Jeffrey, C. (2010) Geographies of children and youth I: eroding maps of life, Progress in Human Geography, 34, 4, 496–505.

[shil13019-bib-0030] Jovchelovitch, S. and Gervais, M.C. (1999) Social representations of health and illness: the case of the Chinese community in England, Journal of Community and Applied Social Psychology, 9, 4, 247–60.

[shil13019-bib-0031] Kelly, P. (2001) Youth at Risk: processes of individualisation and responsibilisation in the risk society, Discourse: Studies in the Cultural Politics of Education, 22, 1, 23–33.

[shil13019-bib-0032] Linell, P. (2009) Rethinking Language, Mind, and World Dialogically : Interactional and Contextual Theories of Human Sense‐Making. Greenwich, Conn.: Information Age.

[shil13019-bib-0033] Lupton, D. (2013) Risk. London, New York: Routledge.

[shil13019-bib-0034] Marková, I. (2003) Dialogicality and Social Representations: The Dynamics of Mind. Cambridge: Cambridge University Press.

[shil13019-bib-0035] Marston, C. , Renedo, A. and Nyaaba, G.N. (2018) Fertility regulation as identity maintenance: understanding the social aspects of birth control, Journal of Health Psychology, 23, 2, 240–51. 28925281 10.1177/1359105317726367 PMC5881789

[shil13019-bib-0036] Miles, S. , Renedo, A. and Marston, C. (2018) Slow co‐production’ for deeper patient involvement in health care, The Journal of Health Design, 3, 1, 57–62.

[shil13019-bib-0037] Miles, S. , Renedo, A. , Augustine, C. , Ojeer, P. , *et al* (2019) Obstacles to use of patient expertise to improve care: a co‐produced longitudinal study of the experiences of young people with sickle cell disease in non‐specialist hospital settings, Critical Public Health, 1–11.

[shil13019-bib-0038] Miller, P. and Rose, N. (1990) Governing economic life, Economy and Society, 19, 1, 1–31.

[shil13019-bib-0039] Morrow, V. (2013) Troubling transitions? Young people's experiences of growing up in poverty in Rural Andhra Pradesh, India, Journal of Youth Studies, 16, 1, 86–100.

[shil13019-bib-0040] National Institute for Health and Clinical Excellence (2012) Sickle cell acute painful episode: management of an acute painful sickle cell episode in hospital: National Institute for Health and Clinical Excellence. 23534086

[shil13019-bib-0041] NICE National Institute for Health and Care Excellence (2016) Transition From Children's to Adults’ Services. Quality standard: National Institute for Health and Care Excellence https://www.nice.org.uk/guidance/qs140/resources/transition-from-childrens-to-adults-services-pdf-75545472790213 (Last accessed 26 August 2019).

[shil13019-bib-0042] Oni, L. , Dick, M. , Smalling, B. and Walters, J . (2006) Care and Management of Your Child With Sickle Cell Disease: A Parent's Guide. London: NHS Sickle Cell and Thalassaemia Screening Programme.

[shil13019-bib-0043] Rabinow, P. and Rose, N. (2016) Biopower Today, Biosocieties, 1, 2, 195–217.

[shil13019-bib-0044] Renedo, A. (2010) Polyphony and Polyphasia in Self and Knowledge, Papers on Social Representations, 19, 12.1–12.21.

[shil13019-bib-0045] Renedo, A. (2014) Care versus control: the identity dilemmas of UK Homelessness professionals working in a contract culture, Journal of Community & Applied Social Psychology, 24, 3, 220–33.

[shil13019-bib-0046] Renedo, A. , Komporozos‐Athanasiou, A. and Marston, C. (2018) Experience as evidence: the dialogic construction of health professional knowledge through patient involvement, Sociology‐the Journal of the British Sociological Association, 52, 4, 778–95.

[shil13019-bib-0047] Renold, E. and Ringrose, J. (2011) Schizoid subjectivities? Re‐theorizing teen girls’ sexual cultures in an era of ‘sexualization’, Journal of Sociology, 47, 4, 389–409.

[shil13019-bib-0048] Rose, N. (1999a) Governing the Soul : The Shaping of the Private Self. London: Free Association Press.

[shil13019-bib-0049] Rose, N. (1999b) Powers of Freedom : Reframing Political Thought, Cambridge. Cambridge: Cambridge University Press.

[shil13019-bib-0050] Salgado, J. and Hermans, H.J.M. (2005) The return of subjectivity: from a multiplicity of selves to the dialogical self, E‐Journal of Applied Psychology: Clinical Section, 1, 1, 3–13.

[shil13019-bib-0051] Tarrant, M. , Hagger, M.S. and Farrow, C.V. (2012) Promoting positive orientation towards health through social identity In Jetten J., Haslam C. and Haslam A. (eds) The Social Cure. Identity, Health and Well‐Being, East. Sussex: Psychology Press, pp 36–54.

[shil13019-bib-0052] Viner, R. (1999) Transition from paediatric to adult care. Bridging the gaps or passing the buck?, Archives of Disease in Childhood, 81, 3, 271–5. 10451404 10.1136/adc.81.3.271 PMC1718076

[shil13019-bib-0053] Wierenga, A. (2011) Transitions, local culture and human dignity: rural young men in a changing world, Journal of Sociology, 47, 4, 371–87.

[shil13019-bib-0054] Wilson, B.H. and Nelson, J. (2015) Sickle cell disease pain management in adolescents: a literature review, Pain Management Nursing, 16, 2, 146–51. 25175555 10.1016/j.pmn.2014.05.015

[shil13019-bib-0055] World Health Organization (2017) Global Accelerated Action for the Health of Adolescents (AA‐HA!): Guidance to Support Country Implementation. Geneva: World Health Organization.

[shil13019-bib-0056] Zittoun, T. (2006) Transitions. Development Through Symbolic Resources. Greenwich: InfoAge.

